# Supplementary material for: National Prevalence of Caprine Prion Protein Genetic Variability at Codons 146, 211, and 222 in Goat Herds in the United States
Source: Vet Sci. 2023 Dec 27;11(1):13. doi: 10.3390/vetsci11010013 (PMC10818752; doi:10.3390/vetsci11010013)
Supplement: Supplementary file 1 [file vetsci-11-00013-s001.zip › vetsci-2711529-supplementary.pdf]

Figure S1. The total number of samples tested per VS district.

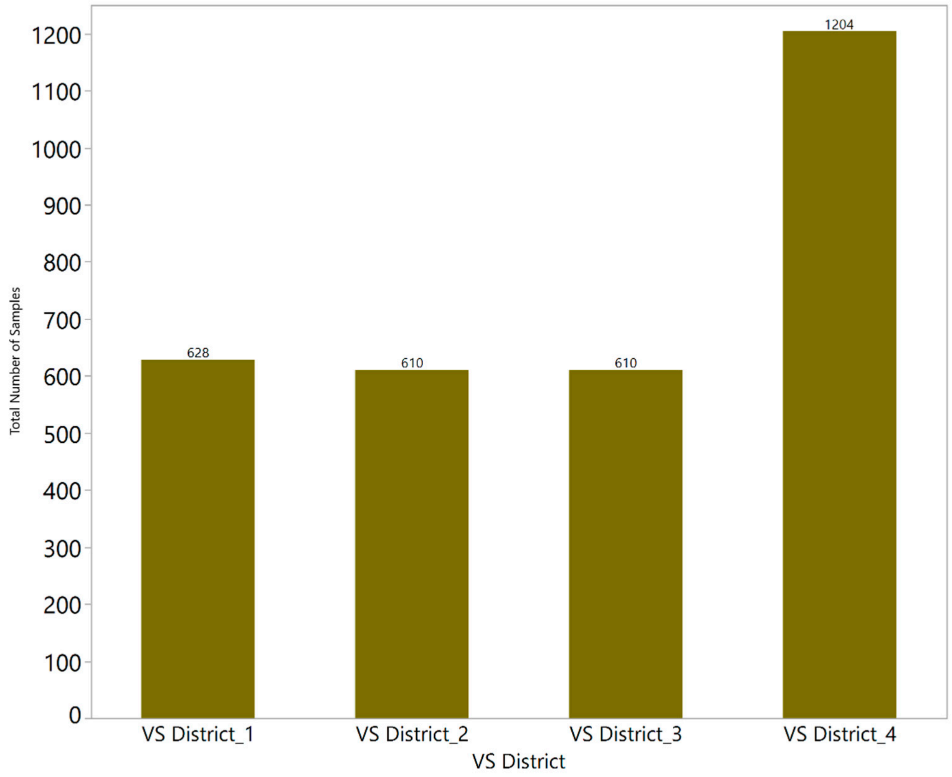

Table S1. Total number and percentage of goats by caprine *PRNP* genotype at codon 146 by state.

State	Total number of samples tested	DD146		DS146		ND146		NN146		NS146		SS146	
		Number of goats	% of Total per state	Number of goats	% of Total per state	Number of goats	% of Total per state	Number of goats	% of Total per state	Number of goats	% of Total per state	Number of goats	% of Total per state
AK	1	0	0.00%	0	0.00%	0	0.00%	1	100.00%	0	0.00%	0	0.00%
AL	53	0	0.00%	0	0.00%	0	0.00%	43	81.13%	9	16.98%	1	1.89%
AR	68	0	0.00%	0	0.00%	1	1.47%	47	69.12%	17	25.00%	3	4.41%
AZ	31	0	0.00%	0	0.00%	2	6.45%	18	58.06%	8	25.81%	3	9.68%
CA	227	0	0.00%	0	0.00%	0	0.00%	157	69.16%	63	27.75%	7	3.08%
CO	58	0	0.00%	0	0.00%	0	0.00%	35	60.34%	20	34.48%	3	5.17%
CT	5	0	0.00%	0	0.00%	0	0.00%	4	80.00%	1	20.00%	0	0.00%
DE	3	0	0.00%	0	0.00%	0	0.00%	3	100.00%	0	0.00%	0	0.00%
FL	71	0	0.00%	1	1.41%	3	4.23%	51	71.83%	13	18.31%	3	4.23%
GA	71	0	0.00%	0	0.00%	0	0.00%	61	85.92%	7	9.86%	3	4.23%
HI	24	0	0.00%	0	0.00%	0	0.00%	18	75.00%	6	25.00%	0	0.00%
IA	94	0	0.00%	0	0.00%	0	0.00%	90	95.74%	4	4.26%	0	0.00%
ID	39	0	0.00%	0	0.00%	0	0.00%	21	53.85%	17	43.59%	1	2.56%
IL	47	0	0.00%	0	0.00%	0	0.00%	38	80.85%	6	12.77%	3	6.38%
IN	62	0	0.00%	0	0.00%	0	0.00%	47	75.81%	12	19.35%	3	4.84%
KS	90	0	0.00%	0	0.00%	0	0.00%	77	85.56%	12	13.33%	1	1.11%
KY	77	0	0.00%	1	1.30%	0	0.00%	67	87.01%	8	10.39%	1	1.30%
LA	30	0	0.00%	0	0.00%	0	0.00%	23	76.67%	5	16.67%	2	6.67%
MA	11	0	0.00%	0	0.00%	0	0.00%	8	72.73%	2	18.18%	1	9.09%
MD	12	0	0.00%	0	0.00%	0	0.00%	9	75.00%	3	25.00%	0	0.00%
ME	8	0	0.00%	0	0.00%	0	0.00%	6	75.00%	2	25.00%	0	0.00%
MI	43	0	0.00%	0	0.00%	0	0.00%	25	58.14%	17	39.53%	1	2.33%
MN	52	0	0.00%	0	0.00%	0	0.00%	45	86.54%	7	13.46%	0	0.00%
MO	158	0	0.00%	0	0.00%	3	1.90%	123	77.85%	26	16.46%	6	3.80%

MS	42	0	0.00%	0	0.00%	2	4.76%	33	78.57%	7	16.67%	0	0.00%
MT	17	0	0.00%	0	0.00%	0	0.00%	13	76.47%	4	23.53%	0	0.00%
NC	58	0	0.00%	0	0.00%	0	0.00%	46	79.31%	12	20.69%	0	0.00%
ND	8	0	0.00%	0	0.00%	0	0.00%	6	75.00%	2	25.00%	0	0.00%
NE	53	0	0.00%	0	0.00%	2	3.77%	36	67.92%	11	20.75%	4	7.55%
NH	5	0	0.00%	0	0.00%	0	0.00%	5	100.00%	0	0.00%	0	0.00%
NJ	9	0	0.00%	0	0.00%	0	0.00%	7	77.78%	2	22.22%	0	0.00%
NM	24	0	0.00%	0	0.00%	0	0.00%	17	70.83%	6	25.00%	1	4.17%
NV	32	0	0.00%	0	0.00%	0	0.00%	27	84.38%	4	12.50%	1	3.13%
NY	32	0	0.00%	0	0.00%	0	0.00%	27	84.38%	4	12.50%	1	3.13%
OH	76	0	0.00%	0	0.00%	0	0.00%	54	71.05%	20	26.32%	2	2.63%
OK	163	0	0.00%	2	1.23%	3	1.84%	102	62.58%	49	30.06%	7	4.29%
OR	69	0	0.00%	0	0.00%	0	0.00%	47	68.12%	21	30.43%	1	1.45%
PA	57	0	0.00%	0	0.00%	1	1.75%	45	78.95%	10	17.54%	1	1.75%
RI	1	0	0.00%	0	0.00%	0	0.00%	1	100.00%	0	0.00%	0	0.00%
SC	49	0	0.00%	0	0.00%	0	0.00%	33	67.35%	11	22.45%	5	10.20%
SD	25	0	0.00%	0	0.00%	0	0.00%	24	96.00%	1	4.00%	0	0.00%
TN	99	0	0.00%	0	0.00%	3	3.03%	74	74.75%	18	18.18%	4	4.04%
TX	600	5	0.83%	4	0.67%	22	3.67%	427	71.17%	122	20.33%	20	3.33%
UT	23	0	0.00%	0	0.00%	0	0.00%	13	56.52%	10	43.48%	0	0.00%
VA	50	0	0.00%	0	0.00%	0	0.00%	38	76.00%	9	18.00%	3	6.00%
VT	11	0	0.00%	0	0.00%	0	0.00%	10	90.91%	1	9.09%	0	0.00%
WA	49	0	0.00%	0	0.00%	0	0.00%	34	69.39%	13	26.53%	2	4.08%
WI	126	0	0.00%	0	0.00%	0	0.00%	116	92.06%	9	7.14%	1	0.79%
WV	23	0	0.00%	0	0.00%	0	0.00%	15	65.22%	7	30.43%	1	4.35%
WY	16	0	0.00%	0	0.00%	0	0.00%	10	62.50%	5	31.25%	1	6.25%

Table S2. Total number and percentage of goats by caprine *PRNP* genotype at codon 211 by state.

State	Total number of samples tested	QQ211		RQ211		RR211	
		Number of goats	% of Total per state	Number of goats	% of Total per state	Number of goats	% of Total per state
AK	1	0	0.00%	1	100.00%	0	0.0%
AL	53	0	0.00%	5	9.43%	48	90.6%
AR	68	0	0.00%	4	5.88%	64	94.1%
AZ	31	0	0.00%	1	3.23%	30	96.8%
CA	227	0	0.00%	8	3.52%	219	96.5%
CO	58	0	0.00%	0	0.00%	58	100.0%
CT	5	0	0.00%	1	20.00%	4	80.0%
DE	3	0	0.00%	1	33.33%	2	66.7%
FL	71	0	0.00%	0	0.00%	71	100.0%
GA	71	2	2.82%	5	7.04%	64	90.1%
HI	24	0	0.00%	1	4.17%	23	95.8%
IA	94	0	0.00%	10	10.64%	84	89.4%
ID	39	0	0.00%	0	0.00%	39	100.0%
IL	47	1	2.13%	5	10.64%	41	87.2%
IN	62	0	0.00%	2	3.23%	60	96.8%
KS	90	0	0.00%	5	5.56%	85	94.4%
KY	77	0	0.00%	2	2.60%	75	97.4%
LA	30	0	0.00%	0	0.00%	30	100.0%
MA	11	0	0.00%	1	9.09%	10	90.9%
MD	12	0	0.00%	0	0.00%	12	100.0%
ME	8	0	0.00%	0	0.00%	8	100.0%
MI	43	1	2.33%	4	9.30%	38	88.4%
MN	52	0	0.00%	2	3.85%	50	96.2%
MO	158	0	0.00%	10	6.33%	148	93.7%
MS	42	0	0.00%	4	9.52%	38	90.5%
MT	17	0	0.00%	1	5.88%	16	94.1%

NC	58	0	0.00%	4	6.90%	54	93.1%
ND	8	0	0.00%	0	0.00%	8	100.0%
NE	53	0	0.00%	0	0.00%	53	100.0%
NH	5	0	0.00%	5	100.00%	0	0.0%
NJ	9	0	0.00%	0	0.00%	9	100.0%
NM	24	0	0.00%	0	0.00%	24	100.0%
NV	32	0	0.00%	0	0.00%	32	100.0%
NY	32	3	9.38%	4	12.50%	25	78.1%
OH	76	0	0.00%	3	3.95%	73	96.1%
OK	163	1	0.61%	3	1.84%	159	97.5%
OR	69	0	0.00%	3	4.35%	66	95.7%
PA	57	0	0.00%	3	5.26%	54	94.7%
RI	1	0	0.00%	0	0.00%	1	100.0%
SC	49	0	0.00%	5	10.20%	44	89.8%
SD	25	0	0.00%	1	4.00%	24	96.0%
TN	99	0	0.00%	2	2.02%	97	98.0%
TX	600	2	0.33%	11	1.83%	587	97.8%
UT	23	0	0.00%	1	4.35%	22	95.7%
VA	50	0	0.00%	2	4.00%	48	96.0%
VT	11	0	0.00%	2	18.18%	9	81.8%
WA	49	0	0.00%	4	8.16%	45	91.8%
WI	126	3	2.38%	20	15.87%	103	81.7%
WV	23	0	0.00%	1	4.35%	22	95.7%
WY	16	0	0.00%	0	0.00%	16	100.0%

Table S3. Total number and percentage of goats by caprine *PRNP* genotype at codon 222 by state.

State	Total number of samples tested	KK222		QK222		QQ222	
		Number of goats	% of Total per state	Number of goats	% of Total per state	Number of goats	% of Total per state
AK	1	0	0.00%	0	0.00%	1	100.00%
AL	53	0	0.00%	0	0.00%	53	100.00%
AR	68	0	0.00%	1	1.47%	67	98.53%
AZ	31	0	0.00%	0	0.00%	31	100.00%
CA	227	0	0.00%	0	0.00%	227	100.00%
CO	58	0	0.00%	0	0.00%	58	100.00%
CT	5	0	0.00%	0	0.00%	5	100.00%
DE	3	0	0.00%	0	0.00%	3	100.00%
FL	71	0	0.00%	4	5.63%	67	94.37%
GA	71	0	0.00%	0	0.00%	71	100.00%
HI	24	0	0.00%	1	4.17%	23	95.83%
IA	94	0	0.00%	1	1.06%	93	98.94%
ID	39	0	0.00%	0	0.00%	39	100.00%
IL	47	0	0.00%	0	0.00%	47	100.00%
IN	62	0	0.00%	0	0.00%	62	100.00%
KS	90	0	0.00%	0	0.00%	90	100.00%
KY	77	0	0.00%	1	1.30%	76	98.70%
LA	30	0	0.00%	0	0.00%	30	100.00%
MA	11	0	0.00%	0	0.00%	11	100.00%
MD	12	0	0.00%	0	0.00%	12	100.00%
ME	8	0	0.00%	0	0.00%	8	100.00%
MI	43	0	0.00%	1	2.33%	42	97.67%
MN	52	0	0.00%	0	0.00%	52	100.00%
MO	158	0	0.00%	2	1.27%	156	98.73%
MS	42	0	0.00%	0	0.00%	42	100.00%
MT	17	0	0.00%	0	0.00%	17	100.00%

NC	58	0	0.00%	0	0.00%	58	100.00%
ND	8	0	0.00%	0	0.00%	8	100.00%
NE	53	0	0.00%	2	3.77%	51	96.23%
NH	5	0	0.00%	0	0.00%	5	100.00%
NJ	9	0	0.00%	0	0.00%	9	100.00%
NM	24	0	0.00%	0	0.00%	24	100.00%
NV	32	0	0.00%	0	0.00%	32	100.00%
NY	32	1	3.13%	1	3.13%	30	93.75%
OH	76	0	0.00%	1	1.32%	75	98.68%
OK	163	0	0.00%	1	0.61%	162	99.39%
OR	69	0	0.00%	0	0.00%	69	100.00%
PA	57	0	0.00%	1	1.75%	56	98.25%
RI	1	0	0.00%	0	0.00%	1	100.00%
SC	49	0	0.00%	1	2.04%	48	97.96%
SD	25	0	0.00%	1	4.00%	24	96.00%
TN	99	0	0.00%	3	3.03%	96	96.97%
TX	600	0	0.00%	7	1.17%	593	98.83%
UT	23	0	0.00%	0	0.00%	23	100.00%
VA	50	0	0.00%	0	0.00%	50	100.00%
VT	11	0	0.00%	0	0.00%	11	100.00%
WA	49	0	0.00%	0	0.00%	49	100.00%
WI	126	1	0.79%	1	0.79%	124	98.41%
WV	23	0	0.00%	0	0.00%	23	100.00%
WY	16	0	0.00%	0	0.00%	16	100.00%
